# Particle Size Determines the Shape of Supraparticles in Self-Lubricating Ternary Droplets

**DOI:** 10.1021/acsnano.0c06814

**Published:** 2021-02-19

**Authors:** Lijun Thayyil Raju, Olga Koshkina, Huanshu Tan, Andreas Riedinger, Katharina Landfester, Detlef Lohse, Xuehua Zhang

**Affiliations:** † Physics of Fluids Group, Faculty of Science and Technology, Mesa+ Institute for Nanotechnology, Max Planck Center for Complex Fluid Dynamics, and J. M. Burgers Centre for Fluid Dynamics, University of Twente, PO Box 217, 7500 AE Enschede, The Netherlands; ‡ Max Planck Institute for Polymer Research, Ackermannweg 10, 55128 Mainz, Germany; § Department of Chemical Engineering, University of California, Santa Barbara, California 93106, United States; ∥ Max Planck Institute for Dynamics and Self-Organisation, Am Fassberg 17, 37077 Göttingen, Germany; ⊥ Department of Chemical and Materials Engineering, University of Alberta, 12-380 Donadeo Innovation Centre for Engineering, Edmonton, T6G1H9 Alberta, Canada; ■ Center for Complex Flows and Soft Matter Research & Department of Mechanics and Aerospace Engineering, Southern University of Science and Technology, Shenzhen 518055, China

**Keywords:** supraparticles, self-lubrication, ternary droplets, evaporation-induced colloidal self-assembly, ouzo effect, silica

## Abstract

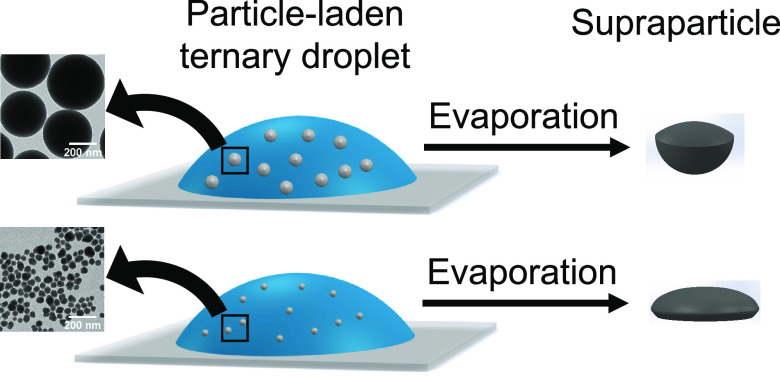

Supraparticles are large clusters of much smaller colloidal particles. Controlling the shape and anisotropy of supraparticles can enhance their functionality, enabling applications in fields such as optics, magnetics, and medicine. The evaporation of self-lubricating colloidal ouzo droplets is an easy and efficient strategy to create supraparticles, overcoming the problem of the “coffee-stain effect” during drop evaporation. Yet, the parameters that control the shape of the supraparticles formed in such evaporating droplets are not fully understood. Here, we show that the size of the colloidal particles determines the shape of the supraparticle. We compared the shape of the supraparticles made of seven different sizes of spherical silica particles, namely from 20 to 1000 nm, and of the mixtures of small and large colloidal particles at different mixing ratios. Specifically, our *in situ* measurements revealed that the supraparticle formation proceeds *via* the formation of a flexible shell of colloidal particles at the rapidly moving interfaces of the evaporating droplet. The time *t*_c0_ when the shell ceases to shrink and loses its flexibility is closely related to the size of particles. A lower *t*_c0_, as observed for smaller colloidal particles, leads to a flat pancake-like supraparticle, in contrast to a more curved American football-like supraparticle from larger colloidal particles. Furthermore, using a mixture of large and small colloidal particles, we obtained supraparticles that display a spatial variation in particle distribution, with small colloids forming the outer surface of the supraparticle. Our findings provide a guideline for controlling the supraparticle shape and the spatial distribution of the colloidal particles in supraparticles by simply self-lubricating ternary drops filled with colloidal particles.

Supraparticles are assemblies of smaller colloidal particles. As a result of the assembly, the supraparticles gain additional functionality as compared to the colloidal building blocks they are made of.^1,2^ In symmetric assemblies, the additional functionality is obtained by a collective response of the colloids, for instance, in optoelectronics, optics, and photonics,^3−7^ catalysis,^8^ and drug delivery.^9^ Tuning the shape and introducing asymmetry and anisotropy in supraparticles can be important in applications where directionality is crucial.^10−25^ Therefore, there is a need for the development of methods that are simple and scalable and that allow to precisely adjust the properties of the supraparticles. Here, we show that changing the size and composition of the colloidal building blocks can tune the shape and anisotropy of supraparticles, made through evaporating self-lubricating ouzo droplets as templates.

The shape and anisotropy of the supraparticles can strongly affect their functionality for applications in a wide range of fields, as shown by a large body of literature.^10−13,21−24^ In the field of optics and optoelectronics, the supraparticle shape can alter the optical response of the supraparticles. For instance, certain nonspherical supraparticles can exhibit multiexciton emission.^12^ Moreover, shape and asymmetry can affect how the supraparticles interact with biological systems. For instance, ellipsoidal supraparticles with sizes in the submicron range were shown to have a lower cellular uptake as compared to spherical supraparticles.^11^ Asymmetric shape of the supraparticles with enhanced surface area was also proposed as desirable in catalyst support.^21^ Finally, supraparticle shape can also play an important role in directed propulsion, since the three-dimensional shape of a microswimmer can influence its dynamical response to an applied field.^13^ A good route for controlling the shape of the supraparticles can be methods that use emulsion droplets or evaporating droplets as templates,^1^ and we explored the second route in this study.

Evaporating droplets as a template for the production of supraparticles offer several advantages.^26,27^ Such droplets can be deployed at precise locations on a surface, thereby enabling to accurately control the position of the produced supraparticles. Moreover, in a certain range smaller than the capillary length, the size of the supraparticles can be tuned easily by controlling the initial size of the droplets. Finally, droplet-based methods can be potentially scaled up in spray drying^4,28^ or in automated droplet generators.^27^ However, the coffee-stain effect—the formation of a ring-like deposit due to the pinned drop boundary and the resulting flow in the drop—represents a major challenge in the application of evaporating droplets as templates.^29^ To overcome this difficulty, self-lubricating droplets have been shown to be an easy way out to circumvent the coffee-stain effect^27^ and to allow for a straightforward production of supraparticles.

To induce self-lubrication, the colloids are first dispersed in an ouzo solution, which is a homogeneous ternary mixture of *trans*-anethole (major component of anise oil), ethanol, and water, the three main ingredients of the Greek aperitif, ouzo.^30−32^ Once a drop of this colloidal ouzo solution is placed on a substrate and the solvents start to evaporate, the higher evaporation rate of ethanol, compared to that of water and oil, reduces the solubility of oil in the droplet. As a result, the oil phase separates and forms a lubricating oil ring at the contact line of the droplet and the substrate.^27,33^ This oil ring prevents the pinning of the contact line and, hence, leads to the formation of a supraparticle instead of a coffee ring.^27^ Thus, the evaporation of colloidal ouzo droplets with their self-lubricating properties is an easy and powerful technique to successfully produce supraparticles.

However, the various factors that affect the properties of the supraparticle formed in self-lubricating ouzo droplets are not yet known. In particular, the effect of the size of colloidal particles on the supraparticle is still unknown. Particle size plays an important role in many colloidal processes such as in the distribution of particles in self-assembled structures and patterns^34−37^ and in agglomeration of the colloids.^38,39^ Moreover, the size of individual colloidal particles can be directly related to the functionality of the resulting supraparticles, for example, in photonic crystals.^1,40^ To explore the effect of the size of the colloidal particles on the properties of the supraparticle, we used silica particles of different sizes as a model system. We have used videography, confocal microscopy, and scanning electron microscopy to explore the supraparticle formation in ouzo droplets.

Our study shows that the size of the colloidal particles determines the shape of the resulting supraparticle. The supraparticle assembly proceeds *via* the formation of a shell made of silica particles at the rapidly moving interfaces of the droplet. The behavior of this particle shell depends on the size of the colloidal particles, which ultimately leads to different shapes of the supraparticles. Combining the effect of size on the supraparticle shape with the size-based stratification of colloids in a mixture of large and small particles,^34,36,41^ we are able to obtain supraparticles with not only nonspherical or asymmetric shapes but also stratified distribution of the colloidal particles inside the supraparticle. These findings can be important in many areas where asymmetry and anisotropy are crucial for the performance of supraparticles, for example, in optics^17,42^ and magnetics^13,18,19^ and in directional propulsion.^20^

## Results and Discussion

### Effect of Colloidal Particle Size on the Shape of Supraparticles

**Figure 1:**
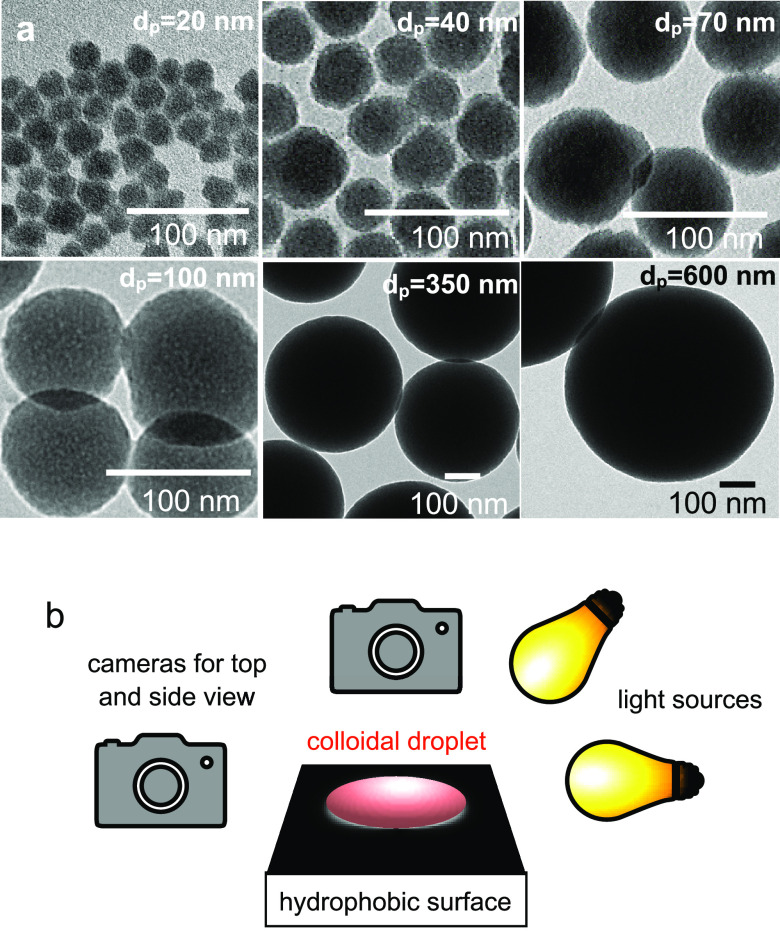
Overview of experimental setup and used colloidal particles. (a) TEM images of different nanoparticles used in this study with approximate particle diameters (*d*_p_) of 20, 40, 70, 100, 350, and 600 nm, respectively (see Table 1). Scale bar: 100 nm. (b) Schematic of evaporation setup. The colloidal droplet was placed on a hydrophobic substrate, and the evaporation process was monitored by a top- and a side-view camera.

To study the effect of colloidal particle size on supraparticle formation in self-lubricating colloidal ouzo droplets, we used monodisperse silica particles of diameters ranging from 20 to 1000 nm (Figure 1a, Table 1). We decided to use silica particles because silica chemistry enables to easily control particle size. Thus, all particles in this study were prepared using the Stöber method, except for the silica particles with 20 nm diameter, for which the lysine method^43^ was used. Moreover, silica nanoparticles are stabilized by their negative charge and do not require any additives which could additionally interact with the oil phase for stabilization. The combination of these properties makes them an ideal model system.

**Table 1:** Characteristics of Nanoparticles Used for Supraparticles Formation^a^

Diameters of the silica particles (nm)	*D* (nm) (TEM)	*D*_h_ (nm) (DLS)	PDI (DLS)	ζ pot. (mV)
20	23 ± 3	29	0.5	–18 ± 1
40	39 ± 8	67	0.1	–40 ± 2
70	71 ± 9	96	0.05	–33 ± 2
100	94 ± 10	126	0.17	–40 ± 1
350	358 ± 26	372	0.04	–54 ± 0.5
600	596 ± 66	600	0.1	–65 ± 0.6
1000	1056 ± 24^b^	1060	0.15	–84 ± 0.5
30 (rhodamine B labeled)	32 ± 6	37	0.2	–21 ± 2
800 (rhodamine B labeled)	776 ± 56	748	0.2	–56 ± 1

a The ζ potential represents the mean and the standard deviation of five independent measurements.

b SEM size. Refer to SI section S12 for SEM images.

Silica particles were dispersed in a homogeneous, clear solution of ethanol, water, and *trans*-anethol to create a colloidal ouzo mixture. While the particle size was systematically varied from 20 to 1000 nm, the initial composition (by weight) of the colloidal ouzo mixture was kept constant at 0.1 wt % silica particle dispersion, 53.2 wt % ethanol, 1.4 wt % *trans*-anethol, and 45.3 wt % water. The choice of the initial composition is based on the previous work by Tan *et al*.^27^ It ensures that (1) the solvents (water and ethanol) and the oil form a single, homogeneous mixture without any phase separation of oil, while the particles are dispersed homogeneously in the ternary mixture, and (2) during the evaporation, an oil ring can form, to enable self-lubrication of the shrinking drop boundary. A ∼1 μL drop of this colloidal ouzo mixture was pipetted on a hydrophobic glass surface, and the evaporation process was simultaneously recorded from the side and the top, using two cameras connected to long-distance microscopes (Figure 1b). As expected, self-lubrication and formation of the oil ring ensured that the contact line does not get pinned for almost the whole duration of the evaporation process. Thus, upon the evaporation of all three volatile liquids, supraparticles are formed in the end.^27^

**Figure 2:**
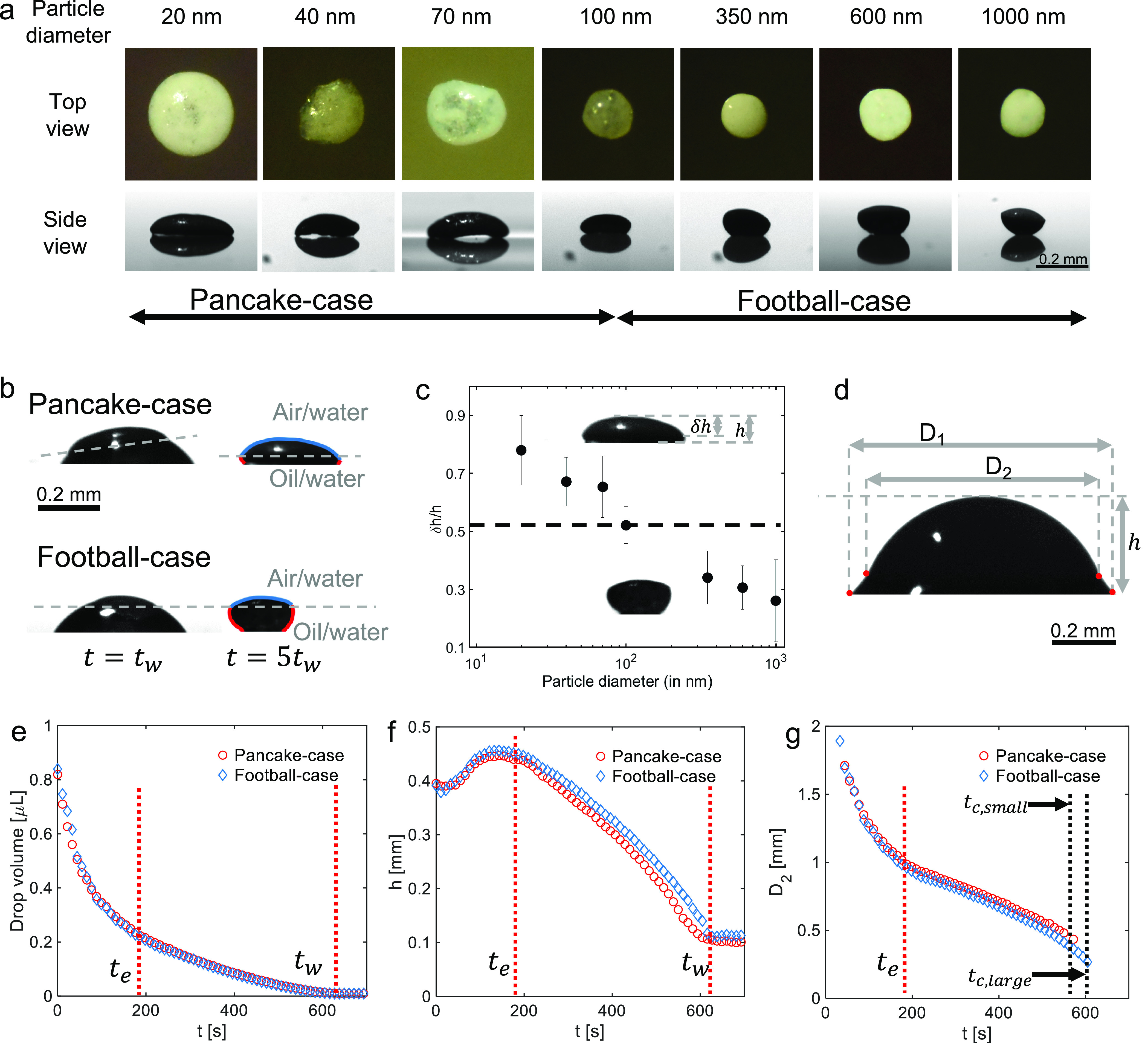
Effect of nanoparticle size on supraparticles formation. (a) Top view (upper row) and side view (lower row) of submillimeter-sized supraparticles obtained after evaporation of colloidal droplets containing silica particles of given sizes. The shape of deposits changes from pancake-like (leftmost) to American football-like (rightmost) with increasing size of the particles (based on side view). (b) Shadowgraph image (side view) of the “American football” shaped and “pancake” shaped supraparticles, at *t* = *t*_w_ (when water has evaporated, but oil is still surrounding the supraparticle) and at *t* = 5*t*_w_ (when also all oil has evaporated). The dotted line demarcates the part of the supraparticle formed at air–water interface (blue-colored) with that formed at oil–water interface (red-colored). The shape of deposits indicates that in the pancake case, there is much less surface formed at the oil–water interface, whereas for the American football case, there is a considerable amount of surface formed at the oil–water interface. (c) *δh*/*h* of the final supraparticles for the different particle sizes. This value is considered as a measure of the part of the surface formed near the air–water interface. *δh*/*h* decreases as the particle size increases, indicating more particles at the oil–water interface for larger particles. 100 nm can be considered as a transition case. (d) A typical shadowgraph image of an ouzo droplet, showing its major geometrical features *D*_1_, *D*_2_, and *h*. (e–g) Drop volume, drop height (*h*), and the estimated oil–water–air contact-line diameter (*D*_2_) of pancake-like (red circles) and American football-like (blue diamonds) cases, plotted *versus* time. Drop volume and *h* have no appreciable differences between the two cases, but *D*_2_ ceases to be distinctly identifiable for the case of smaller particles at *t*_c,small_/*t*_w_ = 0.90 ± 0.01, compared to *t*_c,large_/*t*_w_ = 0.96 ± 0.01 for large particles.

The supraparticles formed after evaporation were different in their shape, depending on the size of colloids (Figure 2a, top view (top row) and side view (bottom row)). In particular, the lateral width of the deposits decreases with increasing particle size (Figure 2a, top view (top row), also see SI Figure S1). Moreover, the side views further reveal a stark difference in the shape of the final supraparticle with increasing particle size. When smaller sized particles with diameters below 100 nm are used, the final supraparticles have a “pancake”-like shape. Oppositely, larger colloidal particles, with diameters above 100 nm, lead to supraparticles with a different shape. For simplicity, we call it an “American football”-like shape, based on the side-view appearance of the supraparticles made of 1000 nm particles (see SI Figure S2 for the statistical margins of the supraparticle shapes in top and side views). Thus, the evaporation experiments show that the size of the colloidal building blocks dramatically affects the shape of the supraparticles.

Furthermore, the different shapes of the supraparticles indicate that the part of the supraparticle surface formed near the air–water interface and the interface between water and the oil ring changes with the size of colloidal particles (Figure 2b). The surface of supraparticles formed near the air–water interface is at the upper part of the supraparticle and is concave downward with a low curvature (Figure 2b, marked in blue). In contrast, the surface of supraparticles formed near the oil–water interface is in the lower part, is concave upward, and has a higher curvature (Figure 2b, marked in red). For pancake-like supraparticles, a greater part of the surface was formed close to the air–water interface (Figure 2b, top row, marked blue) compared to the surface formed close to the lubricating oil–water interface (Figure 2b, top row, marked in red). In contrast, for American football-like supraparticles, there is a considerable part of surface formed near the lubricating oil–water interface (Figure 2b, bottom row, marked in red).

Clearly, the shape of the supraparticle is influenced by the confining oil–water and air–water interfaces. The Neumann triangle is often analyzed to determine the shape of an oil–water–air interface in literature.^44,45^ However, the composition in our evaporating drop changes constantly with time, which makes it challenging to relate the supraparticle shape to Neumann triangles.

To further quantify the differences in the shape of the supraparticles, we determined the ratio between the height of the part of supraparticles that is formed close to the air–water interface (*δh*) and the height of the deposit *h* (Figure 2c). The *δh/h* ratio decreases with increasing size of the colloidal silica particles, confirming the observation that the extent of surface formed close to the air–water interface is larger for smaller silica particles (Figure 2c).

The evolution of the droplet geometry over time shows that the particle size does not have any noticeable effect on the evaporation process (Figure 2d–g; refer to SI section S3 for details of image analysis and section S13 for additional plots, a plot of *D*_1_ and a plot of evaporation rate with time). The droplet volume and the height (*h*) of two typical droplets that contain colloidal silica particles with diameters of either 20 or 1000 nm behave similarly over time (Figure 2e,f). Also, the characteristic time scales *t*_e_ (187 ± 21 s) and *t*_w_ (600 ± 36 s) that describe the duration for almost all ethanol or all water to evaporate, respectively, are similar for both cases (Figure 2e–g, vertical lines). This similarity shows that the particles do not interfere with the evaporation. The evaporation process remains unaffected by the size of the colloidal particles because of their very low weight (0.1 wt % before evaporation starts) for most of the duration of the evaporation process. Moreover, this process is predominately influenced by the liquid components, liquid–liquid phase separation, and the strong Marangoni flow,^46^ which can overcome possible interference of the characteristic time scales from the difference in the volume of different particles in the initial drop.

In contrast, the estimated diameter of the oil–water–air contact line (*D*_2_) evolves differently over time with larger and smaller particles (Figure 2g, *D*_2_ is different from *D*_1_, the diameter of the liquid–solid–air contact line). Specifically, the *D*_2_-ceasing time *t*_c_, which quantifies the time at which *D*_2_ ceases to be distinctly identifiable, is different for larger and smaller particles: Small particles have a *D*_2_-ceasing time *t*_c,small_/*t*_w_ = 0.90 ± 0.01, while for larger particles, a *D*_2_-ceasing time *t*_c,large_/*t*_w_ = 0.96 ± 0.01 is found (Figure 2g, see SI section S3 for details of calculation of *D*_2_). As a result, the final value of *D*_2_ is higher for the droplets with smaller particles compared to the droplet with larger particles (Figure 2g). This behavior seems to result in the increase in lateral width of the final supraparticles as particle sizes decrease.

We first expected that the sedimentation of large particles or the differences in size-dependent advection (movement) of the particles, in response to the fluid flow, is responsible for the formation of the different shapes. However, we could rule out both effects, showing that other factors are responsible for the difference in supraparticle shapes. More details on the effect of sedimentation as well as Stokes number calculations (quantifying particle advection) are presented in the SI sections S4 and S5.

### Flat Supraparticle Shape Results from Lower Ceasing Time (*t*_c0_) of the Shell

To determine the mechanism leading to formation of supraparticles of different shapes, we took a detailed look at the evaporation process using confocal fluorescence microscopy. To that end, large and small rhodamine B-labeled silica particles with corresponding diameters of 30 and 800 nm were synthesized. Furthermore, the oil was stained with perylene.

**Figure 3:**
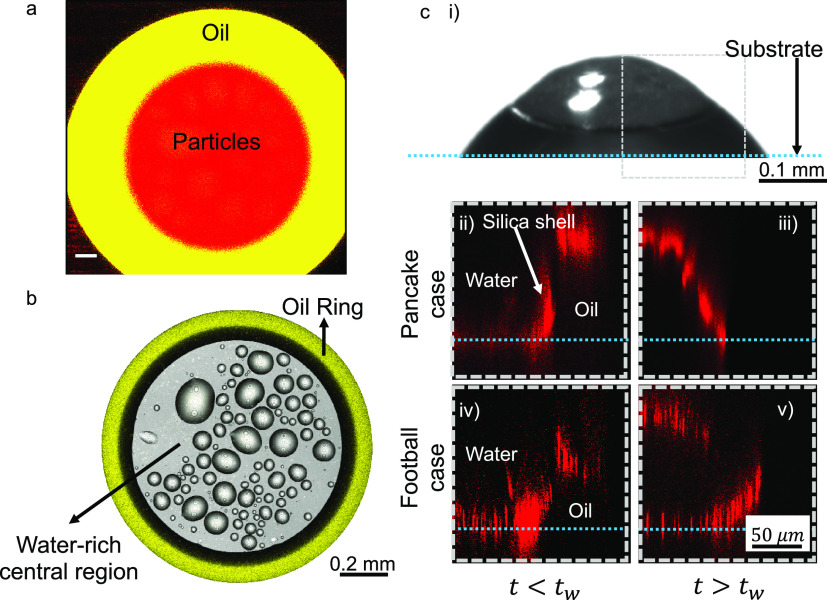
Confocal microscopy of droplet evaporation shows the formation of a shell of silica particles. (a) Confocal microscopy image of evaporating colloidal ouzo droplet shows that most colloids (red) stay in the water-rich central region and not in the oil phase (yellow, perylene). The image corresponds to a horizontal plane close to the substrate (at time *t* = 0.4 × *t*_w_). The ouzo droplet contains rhodamine B-labeled silica particles of 800 nm diameter. Scale bar: 50 μm. (b) An overlay of emission of perylene (yellow, showing oil) and reflection signals shows the interfacial region (black colored) between the oil ring (yellow) and the water-rich central region. The image is taken at a plane very close to the substrate. Scale bar: 0.2 mm. (c) Image of vertical cross-section of droplet using confocal microscopy: (i) Side-view shadowgraph image of droplet at time *t* ≈ *t*_w_. The gray box represents the region in the vertical cross-section in (ii–v). The silica-shell formed at the oil–water interface is similar for both the pancake and the American football cases at *t* < *t*_w_, as shown in (ii) and (iv). Later, at *t* > *t*_w_, the final shape is different as shown in (iii) and (v). Dashed blue lines indicate the position of the substrate. In (ii) and (iii), the ouzo droplet contains smaller particles, 5 wt % 30 nm diameter silica particles (rhodamine B labeled) and 95 wt % unlabeled 20 nm silica particles. In (iv) and (v), the ouzo droplet contains larger particles, 5 wt % 800 nm diameter silica particles (rhodamine B labeled) and 95 wt % unlabeled 1000 nm silica particles.

Fluorescence microscopy confirmed the formation of the oil microdroplets followed by the formation of the oil ring. An overlay of fluorescence emission signals of rhodamine (red) and perylene (yellow) shows that almost all silica particles (red) stay in the water-rich central region of the droplet rather than in the oil-rich droplet-periphery (yellow), for both 30 and 800 nm, rhodamine-labeled silica particles, as expected for hydrophilic particles (Figure 3a). However, the overlay of the emission signal of perylene (yellow) and the reflection signal (greyscale) (Figure 3b) reveals a dark annular region in contact with the yellow-marked oil ring (Figure 3b). This dark region should correspond to a region influenced by the optical effects from the curved surface of the oil ring and by a high concentration of silica particles. The accumulation of silica particles at the oil–water interface of the droplet, forming a shell-like structure around it, is evident from the fluorescence signal of the rhodamine B-labeled silica particles (see SI Figure S5). We refer to this region as “silica shell” further in the text. The oil ring and the silica shell form during the evaporation process for both particle sizes, that is, in the pancake and in the American football cases (Figure 3b and SI Figure S5).

To further understand the shell formation, we took a vertical cross section of the droplet close to the lubricating oil–water interface, reconstructed from the emission signals of rhodamine (Figure 3c, the region observed by confocal microscopy is indicated by a gray box on (i); (ii–v) emission signals from rhodamine B-labeled silica). These results further confirm the formation of a silica shell for both pancake and American football cases. Before water has completely evaporated (*t* < *t*_w_), the shell formation at the oil–water interface proceeds similarly for both cases (Figure 3c(ii,iv)). At the end of the evaporation process, as the time approaches *t*_w_, the upper part of the shell appears. When compared to the side-view shadowgraph images, this upper part should have formed at the air–water interface (Figure 2b, part of the shell marked blue). However, the intermediate steps of the shell formation at the upper part could not be captured. Thus, this upper part of the shell is visible only after it has formed completely (Figure 3c(iii,v)). Finally, the pancake and American football cases have different shapes as shown for *t* > *t*_w_.

**Figure 4:**
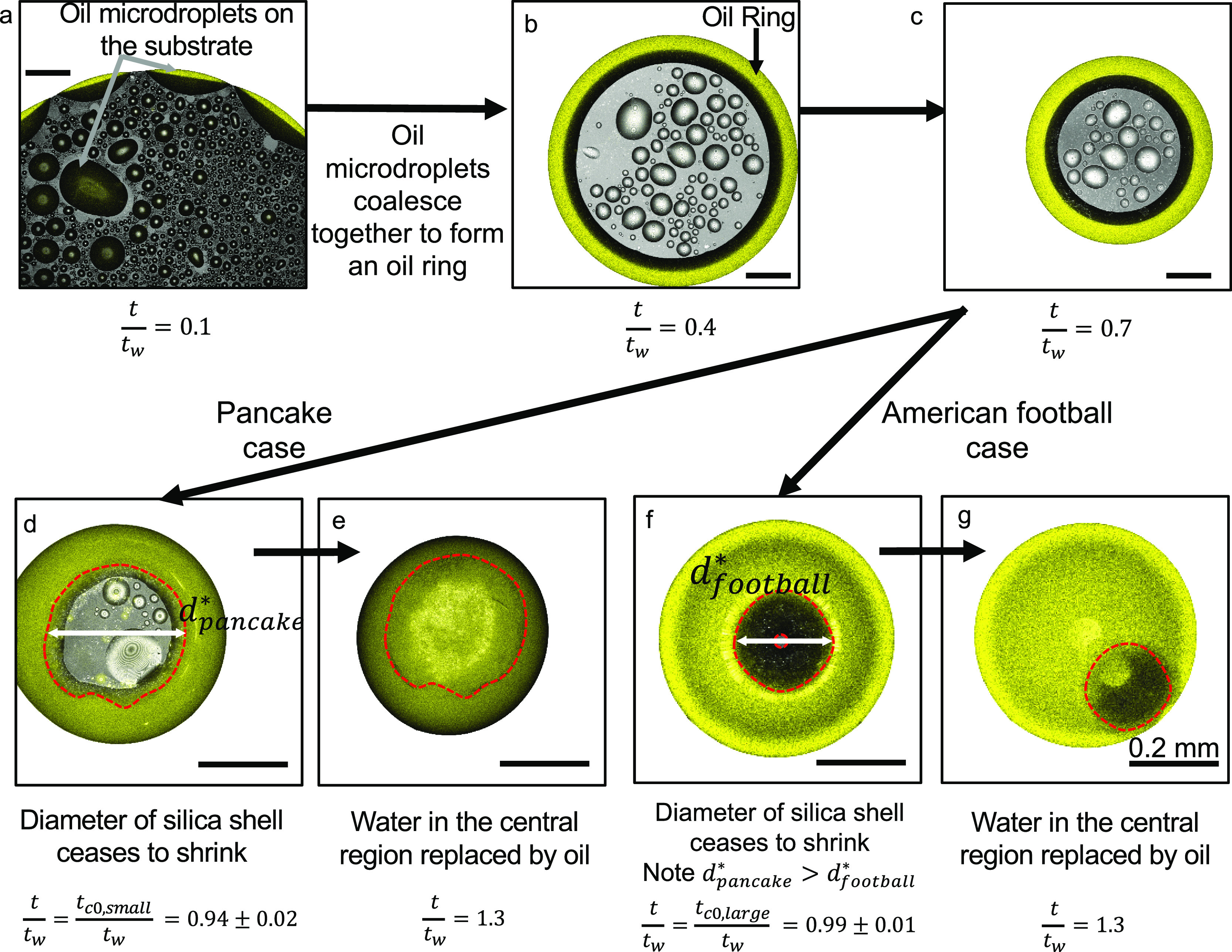
Supraparticle formation visualized by confocal microscopy. An overlay of emission signals of perylene and reflection channels is shown. (a) Initially, the oil microdroplets are formed on the substrate due to phase separation of oil.^46^ (b) Oil microdroplets coalesce, forming the oil ring. (c) The oil ring keeps on contracting in diameter as evaporation of the liquids proceeds. For *t* < *t*_*c*0_, that is, the time before the shell stops contracting, the oil ring contraction is similar for both the pancake and the American football cases. (d–g) At *t* = *t*_c0_, the shell stops contracting. However, *t*_c0,small_ < *t*_c0,large_ and, consequently, the final diameters of the shell are different for the two cases: *d*_pancake_^*^ (for the supraparticle made of smaller particles (d and e)) is larger than *d*_football_^*^ (for the supraparticle made of smaller particles (f and g)). Scale bar: 0.2 mm.

The difference in behavior of larger and smaller particles becomes further evident from the evolution of the oil–water interface where the silica shell exists (Figure 4). Even after the formation of the silica shell, the shell is flexible and keeps on shrinking in lateral width as evaporation proceeds, adjusting its shape to the shape of the oil–water interface (Figure 4a–c, also see in SI Figure S6). However, at a later time, the shell stops contracting (Figure 4d,f). In the case of large particles, the shell deformation ceases at a ceasing time *t*_c0,large_ such that *t*_c0,large_/*t*_w_ = 0.99 ± 0.01, that is, close to the time at which all water has evaporated (Figure 4f). In contrast, with small colloidal particles, the shell ceases deforming at an earlier ceasing time *t*_c0,small,_ such that *t*_c0,small_/*t*_w_ = 0.94 ± 0.02, when water is still present in the central region (Figure 4d). As a result, the base diameter of the pancake-like supraparticle is larger than that of the American football-like supraparticle (*d*_pancake_^*^ > *d*_football_^*^, Figure 4d,f). The ceasing times *t*_c0,large_ and *t*_c0,small_ correspond closely with the *D*_2_-ceasing times *t*_c,large_ and *t*_c,small_, respectively, as observed by videography (see Figure 2g).

After the shell made of small particles stops shrinking (*t*_c0,small_ = 0.94*t*_w_), it cannot conform to the oil–water interface any further. As a result, most of the silica particles accumulate at the air–water interface as water evaporates further, leading to a pancake-like supraparticle in the end (*t* = *t*_w_). In contrast, for larger particles with *t*_c0,large_ = 0.99*t*_w_, the particle shell can conform to the oil–water interface almost until the very end (*t*_w_), leading to considerable amounts of particles at both air–water and oil–water interface. This leads to American football-like supraparticles, with a considerable part of the surface formed near oil–water interface (see Figure 2b for the difference between the two shapes). Finally, for both cases, once *t*_w_ is reached, the oil covers the substrate completely, levitating the supraparticle (Figure 4e,g). Thus, the shape of the supraparticles is determined by the instance when the silica shell ceases to shrink (*t*_c0,large_ and *t*_c0,small_).

Why do the silica particles form a shell near the droplet interface? In evaporating colloidal droplets, the interface of the droplet is moving toward the center of the droplet over the course of evaporation. The formation of a particle shell at the evaporating interface can be caused by an interplay between the motion of the interface and the particle diffusion.^10,47^ This relationship is characterized by the Peclet number *Pe* which is defined as the ratio:
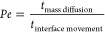
where *t*_interface movement_ is the time scale associated with the motion of the interface and *t*_mass diffusion_ is the time scale associated with diffusion of particles away from the interface, due to Brownian motion. In case where the diffusion is faster than the interface motion (*Pe* ≪ 1), the colloidal particles have sufficient time to diffuse away from the moving interface. Oppositely, if the diffusion is slower (*Pe* ≫ 1), the particles get “caught” by the moving interface and accumulate there, forming a shell.

For our case, with respect to the oil–water interface, we obtain the Peclet numbers which are much larger than unity (*Pe*_o/w,1000_ ∼ 1.6 × 10^3^ for 1000 nm diameter silica particles and *Pe*_o/w,20_∼ 3.2 × 10^1^ for 20 nm diameter silica particles), justifying the formation of a shell (refer to SI section S8, for detailed calculations of the Peclet number). The delayed formation of the shell at the air–water interface could be the result of an interplay of factors such as slightly lower Peclet numbers for the air–water interface (*Pe*_a/w,1000_∼7.6 × 10^2^ for 1000 nm particles and *Pe*_a/w,20_∼1.5 × 10^1^ for 20 nm particles) and the evaporation-induced flow inside the droplet. Specifically, the flow of water that is directed toward the air–water–oil contact line can carry particles away from the air–water interface, hindering the formation of the shell.^48^

One of the reasons for *t*_c0,small_ < *t*_c0,large_ can be due to the dependence of the aggregation time on the particle diameter. The aggregation and gelation time scales, in general, increase with particle size, as the aggregation depends on the number density of particles.^39^ The number density of particles decreases with increasing size for the same mass fraction of particles. Hence, in the case of smaller particles, the higher number of particles leads to a higher rate of aggregation. Furthermore, the size of the colloidal particles also controls the time required for colloidal glass transition.^49^ Such a transition occurs earlier for small colloidal particle aggregates. Similarly, the attractive interaction between accumulated particles and the substrate increases with the number of particles.^50^ Thus, during the final moments before the formation of supraparticles, the smaller particles, with a higher number density, get pinned on the substrate earlier than the bigger particles. Thus, we expect that a combination of the above reasons might be causing the difference between *t*_c0,small_ and *t*_c0,large_, ultimately leading to the two different supraparticle shapes: pancake like or American football like.

In summary, the mechanism of the supraparticle formation in a colloidal ouzo droplet can be described in the following steps: As the solvents in the ouzo droplet evaporate, oil microdroplets form due to phase separation and coalesce together to form a self-lubricating oil ring at the droplet boundary.^27^ As evaporation proceeds, the silica shell initially forms at the oil–water interface and then keeps on adjusting its shape to conform to the shape of the continuously moving drop interface. This silica shell at the oil–water interface becomes rigid at different times for smaller and larger particles (*t*_c0,small_ and *t*_c0,large_*,* respectively). Finally, the silica shell at the air–water interface appears, and the supraparticle formation is complete. The flat shape of the final supraparticle made of smaller particles is due to a smaller *t*_c0,small_*.*

### Bicolloidal Supraparticles with Controllable Shapes and Spatially Varying Composition from Mixed Particles

The goal of this study was to correlate the size of colloidal building blocks and the characteristics of resulting supraparticles to apply the self-lubrication as a route to manufacture functional materials. Therefore, after we revealed the effect of particle size, we next show how to combine this effect with other processes occurring during droplet evaporation to further control the shape of the supraparticles as well as the distribution of particles within the supraparticles. One of processes that is especially interesting in our case is the size-based segregation and stratification of colloids when a mixture of two different sizes is used. This effect was described recently for thin films^41^ and droplets that were either levitated^36^ or placed on superamphiphobic surface.^34^ The size-based stratification happens at high Peclet numbers,^34,41^ as is the case in our system. The shell formation at the moving interface leads to an osmotic gradient within the droplet, which is steeper for larger particles.^34,41^ As a result, small colloids form a layer on top of the larger colloids.

**Figure 5:**
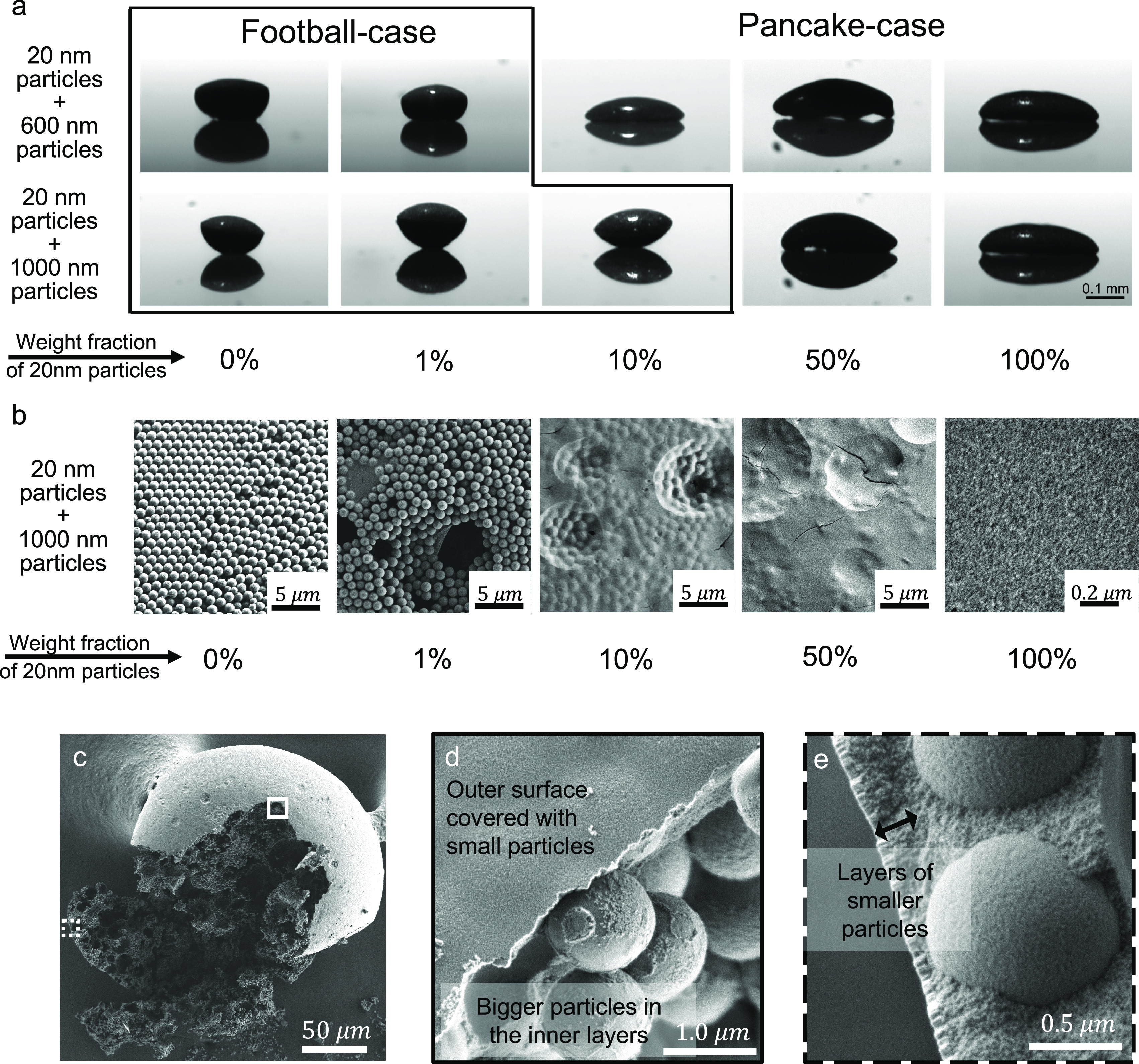
Formation of supraparticles using a mixture of silica particles of two sizes. (a) The shape of supraparticles can be tuned between American football like and pancake like by adding 20 nm nanoparticles to 600 nm (upper row) or 1000 nm (lower row) particles. Scale bar: 0.1 mm. (b) SEM images of the surface of supraparticles with different weight fraction of 20 nm nanoparticles reveal different surface compositions. With the increasing weight fraction of 20 nm nanoparticles, smaller nanoparticles tend to accumulate at the surface. (c) SEM image of a typical stratified supraparticle made of a mixture of 20 and 1000 nm diameter silica particles (with weight fraction of 20 nm particles being 10%), split in the middle to view distribution of particles. The outer surface of the supraparticle is covered with small particles. The regions marked by square and dotted square are shown with more details in (d) and (e), respectively. (d) Magnified SEM image of the square marked region of the supraparticle shown in (c). The top left part shows that smaller particles completely cover the outermost surface, while the lower right part shows the presence of larger particles in the inner layer. (e) Zoom-in on the dotted square cross-sectional region in (c). The region close to the outer surface, also marked by a double-sided arrow, has layers of 20 nm particles.

We investigated supraparticle formation in bicolloidal droplets, using mixtures of larger silica particles (having 600 nm diameter or 1000 nm diameter) with the 20 nm diameter silica nanoparticles (Figure 5a). The weight fraction of the smaller silica particles is reported with respect to the overall weight of silica particles in the ouzo droplet. From shadowgraph images, it becomes evident that mixing of larger and smaller particles at different mixing ratios leads to supraparticles with different shapes. At higher weight fractions of larger particles, the supraparticles display the American football shape. The shape gradually changes toward a pancake-like shape with an increasing content of smaller particles (Figure 5a). The amount of small particles in the mixture that is needed to shift the shape from American football shape to pancake-like shape is higher for 1000 nm silica particles compared to 600 nm silica particles (Figure 5a), underscoring the higher tendency of larger particles to form American football-shaped supraparticles. Thus, mixing the large and small particles at different ratios enables to further fine tune the shape of supraparticles.

To resolve the distribution of particles in the supraparticle, we performed scanning electron microscopy (SEM, Figure 5b–e, also see SI Figure S8). SEM reveals that with increasing weight fraction of smaller particles, not only the shape of supraparticle but also the composition of its surface changes (Figure 5b). In particular, the surface of supraparticles with *w*_SiO_2__ = 1% consists mostly of 1000 nm particles. When the weight fraction of 20 nm nanoparticles increases to 10% or 50%, 20 nm nanoparticles cover the outer surface of the supraparticle (Figure 5b). To further study the distribution of the particles of both sizes within the supraparticles, we split a supraparticle in the middle and looked at the cross-section (Figure 5c). SEM of the cross-section confirmed that the regions close to the outer surface are enriched with 20 nm particles (Figure 5d,e; refer to SI section S11 for more SEM images), though, in our case, we could not obtain full segregation of the different sizes. This process may be further regulated by increasing the Peclet number, for example, by altering humidity and correspondingly the evaporation rate.

The SEM images (Figure 5c, SI Figure S9c,d) also indicate that the supraparticles have a porous internal structure, suggesting suitability in applications where a large surface area is desirable. This structure is in accordance with our earlier study^27^ where the supraparticles obtained by self-lubrication possess a porous and fractal-like structure, analogous to the high porosity of xerogel.^51^ We further measured the mechanical strength of the supraparticles using a compression testing setup.^52^ The mechanical strength measurements show that the fracture force for supraparticles made of 1000 nm silica particles is 1.5 ± 0.3 mN, while the fracture force for supraparticles made of 20 nm silica particles is 0.8 ± 0.5 mN (see SI section S14). The differences in mechanical strength of these two types of supraparticles can either arise from differences in the particle-size-dependent interparticle interactions for the two cases or the geometrical shape and the internal structure of the supraparticles.^53−55^ We also show that the supraparticles are stable upon resuspension in water and ethanol (see SI section S9, SI Videos V1 and V2). Such a large amount of supraparticles can also be collected by evaporating a myriad of droplets deposited on a large flat substrate by inkjet printing.

These findings on the effect of particle size can be combined with other effects to further control the shape of the supraparticles in the future. In particular, it would be interesting to study the additional role of the ratio of volumes of oil and particles in determining the supraparticle shape, along the lines of Tan *et al*.,^27^ and how this ratio interferes with the effect of colloidal particle size. Furthermore, a question to be addressed is whether our findings can be generalized and applied to the production of supraparticles on other substrates, such as structured superhydrophobic surfaces or oil-coated surfaces, and whether the pinning can be avoided completely or for a major part of the evaporation process on structured surfaces.^10,26,56−58^ As the shape of the supraparticle has been leveraged for desirable functionality of the supraparticle in the extensive literature,^11−13,21−24^ it will also be exciting to explore the functionalities of these supraparticles in the future.

## Conclusion

Supraparticles have an enormous potential in a wide variety of fields such as optics, magnetics, catalysis, and biomedical applications. Self-lubricating colloidal droplets are a promising technique to produce supraparticles. In this work, we have shown that the size of the colloids determines the shape of supraparticles that are made using sessile self-lubricating ternary colloidal ouzo droplets. Large silica particles lead to American football-shaped supraparticles, while smaller ones lead to pancake-shaped supraparticles. We demonstrate that the supraparticle formation proceeds *via* formation of a shell of silica particles at the rapidly moving interfaces owing to the high Peclet numbers in the system. The size of the colloids determines the behavior of this particle shell, which in turn affects the shape of the final supraparticle. Furthermore, we made use of the phenomenon of stratification of colloids of different sizes, which occurs due to an osmotic pressure gradient built up during the evaporation of colloidal droplets with particles of different sizes. Thereby, using a mixture of large and small colloidal particles in a single droplet, we produced supraparticles with a distinct layer of small particles on the outer surface. Thus, this work also describes the formation of nonspherical and asymmetric supraparticles that have a spatially varying particle distribution and are made from evaporating self-lubricating ternary droplets filled with dispersed colloidal particles of different sizes. By using other nanoparticles and varying their sizes, our approach can be potentially used for the production of functional materials.

## Materials and Methods

### Materials

Following chemicals were used as received: Tetraethoxysilane (TEOS), trichloro(octadecyl)silane (OTS, ≥90%), hexadecane (99%), and absolute ethanol for synthesis of the nanoparticles, *trans*-anethole (99%) from Merck/Sigma-Aldrich and ethanol for droplet evaporation experiments from Boom BV (100% (v/v), technical grade), ammonium hydroxide (28%) from Fluka, (3-aminopropyl) triethoxysilane (APTES) from TCI chemicals, and 1.0 μm diameter SiO_2_ from monodisperse Particles Pty Ltd., Australia. Milli-Q water was produced by a Reference A+ system (Merck Millipore) at 18.2 MΩ cm at 25 °C for droplet evaporation experiments. For particle synthesis, water that was purified using a Millipore Synergy System. The commercial nanoparticles that were used (see figures in SI) were titanium(IV) oxide (Aldrich, nanopowder, 21 nm, ≥99.5%), silicon dioxide (Aldrich, nanopowder, 10–20 nm, ≥99.5%), and tin(IV) oxide (Aldrich, nanopowder, ≤100 nm avg. part. size). Before using these commercial nanopowders, they were heated at 400 °C for 1 h (to remove possible contaminants).

### Preparation of Nanoparticles

Nanoparticles of 20 nm diameter were synthesized using an amino acid method.^43^ Lysine monohydrate (1.03 g, 6.2 mmol) was dissolved in ultrapure water (205 g), and the solution was heated to 60 °C. TEOS (5.3 mL) was added carefully through a syringe so that it stayed on top of the aqueous phase. The resulting two-phase solution was stirred at 150 rpm using a 2.5 cm stir bar for 2 days. Afterward, cyclohexane was added carefully on top of the aqueous phase until a layer of cyclohexane was visible, to prevent mixing of nonreacted TEOS with the dispersion of nanoparticles. The aqueous phase was taken out carefully with a needle and purified by dialysis in ultrapure water.

All other silica nanoparticles (TEM-sizes of 40 to 600 nm) were prepared using variations of the Stöber method.

For synthesis of nanoparticles having diameter of 70 nm, TEOS (4 mL) was added to a solution of ammonium hydroxide (4 mL, 28% aqueous solution) in absolute ethanol (50 mL) and sonicated in an ultrasonic bath for 2 h, at 36 °C.^59^ The nanoparticles were purified by dialysis in ultrapure water and filtered through a paper filter.

Nanoparticles with 40 nm diameter were prepared by a slight modification of the above method, using ammonium hydroxide (3 mL, 28%) and TEOS (4 mL). After the sonication, the dispersion was stirred overnight and purified by dialysis in ultrapure water.

For synthesis of nanoparticles 100 nm particles, TEOS (3 mL) was dissolved in absolute ethanol (80 mL). This solution was added dropwise to a mixture of ammonium hydroxide (28%, 3 mL), ethanol (20 mL), and water (0.5 mL). The reaction mixture was stirred overnight, centrifuged for 10 min at 16000 *g* to isolate the nanoparticles that were then resuspended in water, and further purified by dialysis in ultrapure water.

The silica particles with 350 nm diameter were prepared by adding TEOS (6.2 mL) to a mixture of ammonium hydroxide (2.34 mL), water (11 mL), and ethanol (80.5 mL) with a syringe pump within 30 min. After stirring overnight, nanoparticles were purified the same way as the 100 nm diameter particles.

The silica particles with 600 nm diameter were synthesized by adding TEOS (7 mL) to a solution of ammonium hydroxide (16 mL, 28%) to absolute ethanol (200 mL) and ultrapure water (11 mL).^43^ After stirring overnight at room temperature, particles were isolated by centrifugation at 16 kg for 15 min. The pellet was resuspended in ultrapure water, and nanoparticles were purified by dialysis.

The rhodamine B-APTES conjugate was synthesized similar to literature procedure.^60^ Rhodamine B isothiocyanate (10 mg, 0.018 mmol) and APTES (40 μL, 37.8 mg, 0.17 mmol) were dissolved in 3 mL of absolute ethanol and stirred overnight under Ar. The resulting solution of dye was stored at 4 °C and used for synthesis of nanoparticles without further purification.

Rhodamine B-labeled 800 nm particles were synthesized using the same method as the nonlabeled particles 600 nm particles. The stock solution of rhodamine B-APTES conjugate (100 μL, corresponds to approximately 6.1 × 10^–4^ mmol rhodamine) was premixed with TEOS prior to the addition of TEOS to the solvent.

Synthesis of rhodamine B-labeled small particles with a diameter of 30 nm was performed similar to nonlabeled nanoparticles of 20 nm with some variations. The rhodamine B-APTES conjugate (382 μL) was first dried on rotary evaporator to remove ethanol and subsequently resuspended in TEOS. The resulting pink solution was added on top of aqueous phase. After stirring for 2 days at 60 °C and purification by dialysis, particles were additionally filtered through a folded filter.

### Dynamic Light Scattering

The DLS was done with a Malvern Zetasizer Nano series S90 at a scattering angle of 90°. Nanoparticles were diluted in ultrapure water so that the attenuator was at step 9–11. The data analysis was performed automatically by Malvern Software v 7.12. The reported values represent the *z*-average obtained from the cumulant analysis. The reported values represent a mean of four independent measurements.

### ζ Potential

The ζ potential was measured with a Malvern Zetasizer Z in disposable ζ potential cuvettes from Malvern using 3 mM KCl as a dispersant. The ζ potentials were calculated by Malvern software from electrophoretic mobility using the Smoluchowski equation. The reported values represent a mean of five independent measurement and the standard deviation.

### Transmission Electron Microscopy

TEM was done at JEOL1400 microscope at an acceleration voltage of 120 kV. The samples were deposited on carbon-coated copper grids. The analysis of particles sizes was done with Fiji using the analyze particles plug in (the particles were separated by watershed filter after setting the threshold). At least 30 particles were measured for each sample.

### Scanning Electron Microscopy

SEM of 1000 nm particles was acquired with a LEO Gemini 1530, landing voltage (EHT) 3 kV. Aqueous dispersion of supraparticles was drop casted on Si-wafer (as deliv., PLANO-EM#G3390). SEM of the supraparticles was done with a Zeiss MERLIN HR-SEM.

### Substrate Preparation

Glass substrates were made hydrophobic by the method of dip coating.^27^ The glass substrates were first wiped with ethanol-wetted tissue. Thereafter, the substrates were rinsed with ethanol and water. Then they were sonicated in acetone, ethanol, and water successively for 5 min each. This process of cleaning by sonication was repeated one more time. Thereafter, the glass substrates were dried carefully using a stream of nitrogen gas. The glass slides were finally subject to aggressive cleaning and oxidation either by plasma cleaning or dipping in piranha solution for 30 min. Thereafter, the dry glass substrates were dipped in a 0.4% (v/v) solution of trichloro(octadecyl)silane (OTS) in hexadecane (the substrates have to be cleaned in water and dried in vacuum before dipping in OTS solution if piranha was used). After 20 min, these substrates were put in a solution of chloroform for another 15 min to remove all nonbound OTS from the surface. The substrates were finally rinsed with ethanol and water to remove any traces of the chloroform and dried with nitrogen before using the glass substrates. The substrates were characterized by contact angles measurements of a water droplet on the hydrophobic substrates. The substrates had an advancing contact angle of 113° ± 2° and receding contact angle of 102° ± 3°.

### Solution Preparation for the Drop Evaporation

The colloidal ouzo solution was prepared by mixing the chemicals in the following order: silica particles (0.1 wt %; silica particles are in concentrated aqueous dispersion), ethanol (53.2 wt %), oil (1.4 wt %), and water (45.3 wt %). The mixture was sonicated in an ultrasonic bath each time for at least 5 min before adding the next chemical.

### Experimental Setup Details

The evaporation of droplets was recorded from the side using a monochrome 8-bit CCD camera (XIMEA, MD061MU-SY, 2752 × 2202 pixel resolution, 1 frame/s) connected to a Navitar 12× adjustable zoom lens. The drop was also monitored from the top using a CMOS color camera (Nikon D750, 1920 × 1080 pixel resolution, 24 frames/s) connected to a similar Navitar 12× adjustable zoom lens. The evaporating drops were illuminated by using LED light sources. The ambient temperature and humidity were measured using a thermo-hygrometer (OMEGA; HH-USD-RP1). The relative humidity was measured to be 45 ± 10%, while the temperature was measured as 20.5 ± 0.5 °C.

### Confocal Microscopy

Nikon Confocal Microscopes A1 system (with 10× and 20× dry objectives) and Leica SP8 (10× water immersion objective) were used for performing confocal microscopy.

### Image Analysis

MATLAB-R2019 and FIJI^61^ were used to analyze the images obtained using confocal microscopy and side-view shadowgraphy.

## Supporting Information Available

Brief discussions on characterization of supraparticle, more images of supraparticles to show repeatability, analysis of shadowgraph images, role of sedimentation in supraparticle formation, Stokes number calculations, formation of silica shell close to oil–water interface, contraction of silica shell, Peclet number calculations, stability of supraparticles, SEM images of supraparticles, SEM images showing smaller particles on the outer surface of bicolloidal supraparticle, SEM characterization of silica 1000 nm particles, further plots describing droplet evaporation, and mechanical stability of supraparticles (PDF)
Video V1: Video showing stability of supraparticle even after redispersion (MP4)
Video V2: Video showing stability of supraparticle even after redispersion (MP4)
Video V3: Video of evaporating droplet as viewed using confocal microscopy (MOV)

## Supplementary Material

nn0c06814_si_003.mp4

nn0c06814_si_004.mov
