# Supplementary material for: Particle Size Determines the Shape of Supraparticles in Self-Lubricating Ternary Droplets
Source: ACS Nano. 2021 Feb 19;15(3):4256–67. doi: 10.1021/acsnano.0c06814 (PMC8023807; doi:10.1021/acsnano.0c06814)
Supplement: Supplementary file 1 — nn0c06814_si_001.pdf [file nn0c06814_si_001.pdf]

Supporting information to

Particle Size Determines the Shape of Supraparticles in Self-Lubricating Ternary Droplets

Lijun Thayyil Raju[1#], Olga Koshkina[2#], Huanshu Tan[3,4], Andreas Riedinger[2], Katharina Landfester[2]*, Detlef Lohse[1,5], Xuehua Zhang[6,1]*

[1]Physics of Fluids Group, Faculty of Science and Technology, Mesa+ Institute for Nanotechnology, Max Planck Center for Complex Fluid Dynamics, and J. M. Burgers Centre for Fluid Dynamics, University of Twente, PO Box 217,7500 AE Enschede, The Netherlands

[2]Max Planck Institute for Polymer Research, Ackermannweg 10, 55128 Mainz, Germany

[3]Center for Complex Flows and Soft Matter Research & Department of Mechanics and Aerospace Engineering, Southern University of Science and Technology, Shenzhen 518055, China

[4]Department of Chemical Engineering, University of California, Santa Barbara, CA 93106, United States

[5]Max Planck Institute for Dynamics and Self-Organisation, 37077 Göttingen, Am Fassberg 17, Germany

[6]Department of Chemical and Materials Engineering, University of Alberta, 12-380 Donadeo Innovation Centre for Engineering, Edmonton, T6G1H9 Alberta, Canada

*Corresponding Authors:

xuehua.zhang@ualberta.ca, landfester@mpip-mainz.mpg.de

[#] LTR and OK contributed equally to the work

Table of Content

S1. Characterization of Supraparticles 3

S2. Supraparticles obtained with nanoparticles of different sizes 4

S3. Analysis of shadowgraph images 5

S4. The role of sedimentation in supraparticle formation 7

S5. Calculations of Stokes numbers 8

S6. Formation of silica shell close to oil-water interface 10

S7. Contraction of silica shell 10

S8. Peclet number calculations 11

S9. Stability of supraparticles 12

S10. SEM images of supraparticles 14

S11. SEM images showing smaller particles on the outer surface of bicolloidal supraparticle 15

S12. Characterization of 1000 nm silica particles by SEM 16

S13. Plots of droplet evaporation 17

S14. Mechanical stability of supraparticles 18

REFERENCES 19

S1. Characterization of Supraparticles

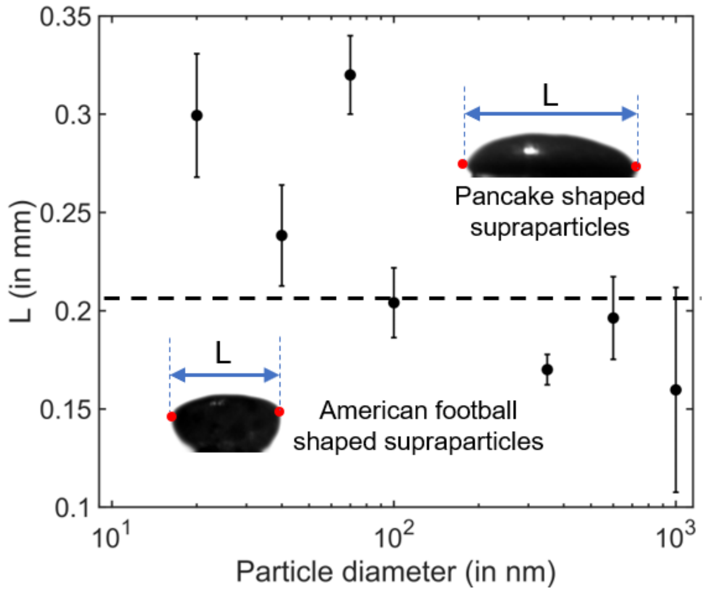

Figure S1. Plot showing decrease in the lateral width of the supraparticles as particle size increases. The two points (marked in red) correspond to the location at which there is a large change in curvature. These points are manually selected for all the supraparticles.

S2. Supraparticles obtained with nanoparticles of different sizes

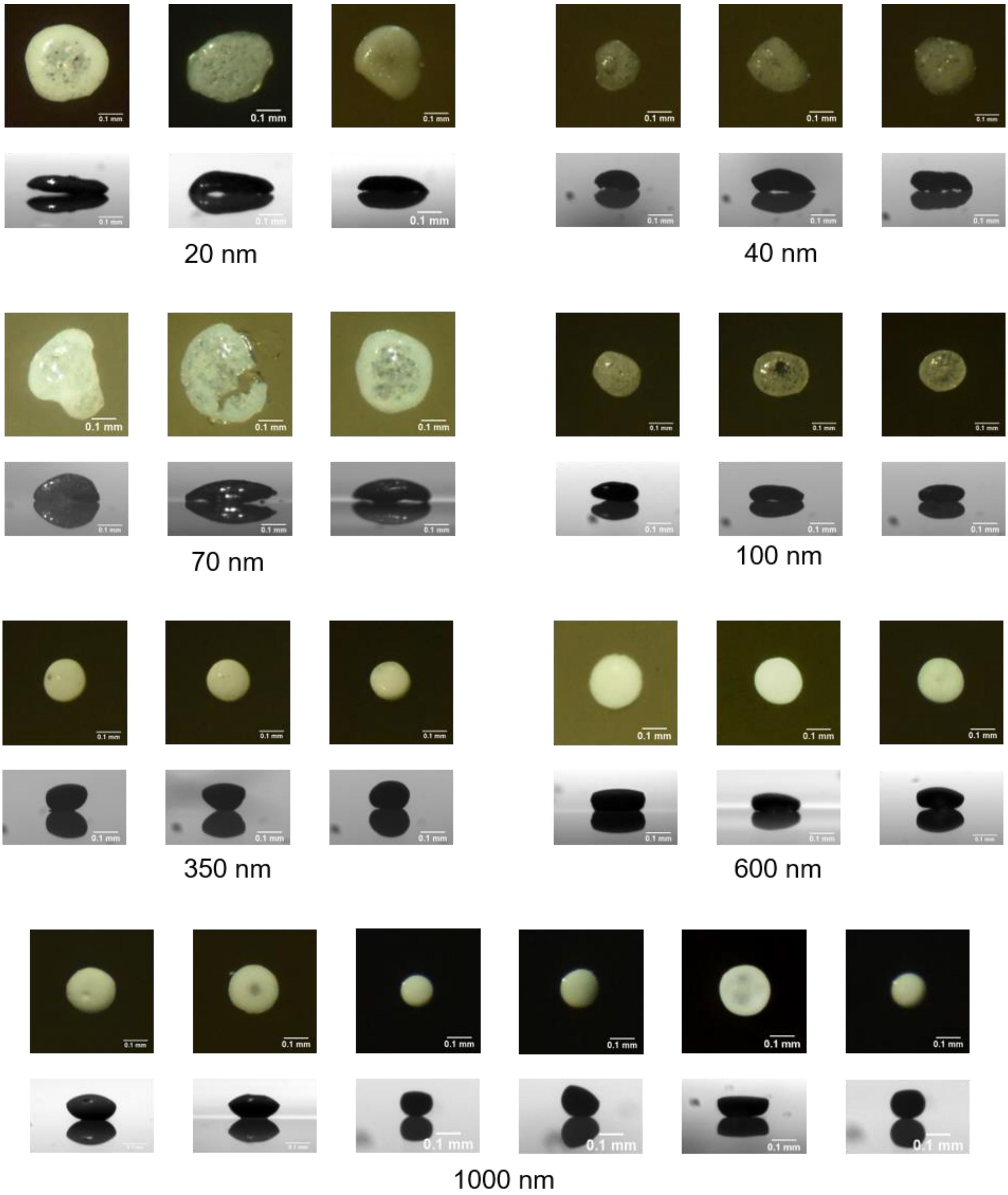

Figure S2. Top view (upper rows) and side view (lower rows) of sub-millimeter sized supraparticles obtained after evaporation of colloidal droplets containing silica particles of given sizes (20 nm to 1000 nm), showing the statistical variations in the supraparticle shape. Scale bar: 0.1 mm

S3. Analysis of shadowgraph images

To study the evolution of the drop geometry over time, the greyscale sideview images recorded using the 8-bit CCD camera (XIMEA) were processed using FIJI and MATLAB to detect the drop edges and to approximate the drop edges by a smooth contour (Figure S3, red line). The measurements were started ~20s after the drop is deposited on the surface.

Measurement of drop volume

The drop volume was calculated by using the detected drop contour, assuming axial symmetry and numerically evaluating the following integral (using the trapezoidal method, using MATLAB)

$$V = \int_{y=y_{min}}^{y=y_{max}} \pi r^2 \, dy$$

where r is the distance of the edge of the droplet from the axis of symmetry (defined as the mid-point of the two points where oil-air-solid contact occurs), for a given y.

The volume is calculated using the contours on both sides of the axis of symmetry and a mean of the two values is used.

Measurement of D_2 (an estimate of oil-water-air contact line)

The oil-water-air contact line is estimated by finding the point on the drop contour where the second derivative (d^2y/dx^2) reaches a minimum (Figure S3, marked by red circles). This corresponds to the point on the drop contour, where the slope changes very steeply. To avoid spurious detections, a minimum threshold value of d^2y/dx^2 (in magnitude) is chosen and any minima of d^2y/dx^2 too close to the center is avoided. The same threshold is used for all the cases to ensure uniformity in detection criteria.

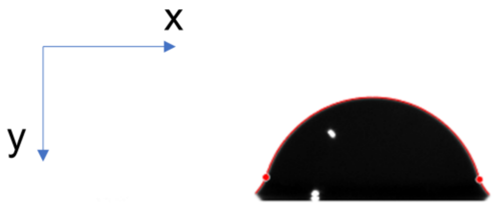

a

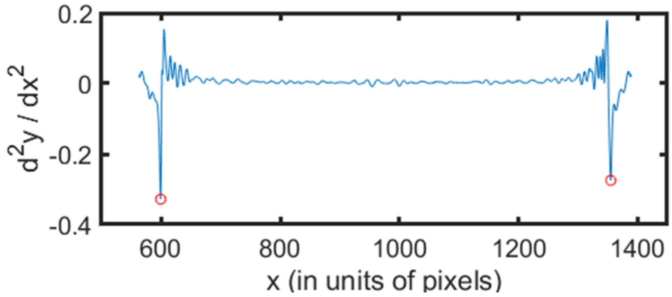

b

Figure S3. Processing of shadowgraph image. (a) Typical side-view image of the evaporating droplet. The red line shows the detected drop edge. The two circles show the estimated location of the oil-water-air contact line. (b) Graph showing the variation of the second derivative (d^2y/dx^2) of the drop. The location of the oil-water-air contact line is estimated by the two minimas of the curve, to the left and right of the axis of symmetry (marked by red circles). They the same points as in (a).

S4. The role of sedimentation in supraparticle formation

In drying colloidal droplets, an interplay between evaporative flux and sedimentation often governs the shape of the final supraparticles. Therefore, we first expected that the sedimentation of large particles might be responsible for the American-football shaped supraparticles in our case. To test this, we performed the evaporation experiments with 1000 nm silica particles, and turned the substrate upside-down right after depositing the droplet. The resulting supraparticles (Figure S4: both top rows) had similar shape as the ones that were obtained while drying in the normal straight position (Figure S4: both bottom rows). Thus, sedimentation is not the driving force that causes the difference between supraparticles from larger or smaller nanoparticles. The minor variation in the shape of the supraparticle between the supraparticles made from upside down and upright orientations (please refer Figure S4 as well as Figure S2-bottom row) appears to be experimental variations only.

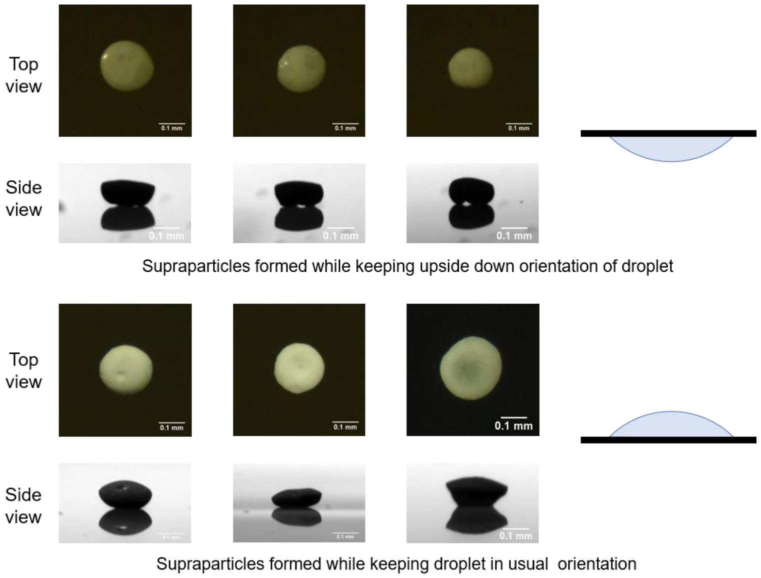

Figure S4. Top-view and side-view images showing that sedimentation is not the driving force for the difference between supraparticles from larger or smaller nanoparticles. Sideview shadowgraph images of American-football shaped supraparticles obtained for different orientations of the droplet: (upper two rows) droplet kept upside down; (lower two rows) droplet placed in the usual orientation.

S5. Calculations of Stokes numbers

In drying colloidal droplets, the response of particles to the fluid flow can be dependent on the size of the particles. Therefore, we considered an option that larger particles do not move with the flow field of the droplet-fluid during evaporation, while the smaller particles do. This behavior can be studied by looking at the Stokes number for the system. Stokes number is defined as the ratio of the characteristic relaxation time scale of particle in response to the drag due to the flow field ($t_{relaxation}$), to the characteristic time scale of the flow (t_{flow}).

$$St = \frac{t_{relaxation}}{t_{flow}}$$

$$t_{relaxation} = \frac{2}{36}\frac{\rho d^2}{\mu}$$

where ρ is the density of the particles, d is the diameter of the particles and μ is the viscosity of water.

Using $\rho = 1.9\ g/cm^3$ and $\mu = 0.9895\ mPa\ s$, we get

- For $d \sim 20nm$, $t_{relaxation} \sim 10^{-11}\ s$
- For $d \sim 1000$nm, $t_{relaxation} \sim 10^{-7}\ s$

If we assume the supra-particle formation happens only after the intense Marangoni flow has subsided, we can approximate $t_{flow} = l/u_{flow}$, where $l \sim r_{base} \sim 0.5\ mm$ and $u_{flow} \sim 50 \mu m/s$,[1] then $t_{flow} \sim 10s$

Thus, the Stokes number for the 20 nm and 1000 nm diameter particles are $St_{20nm} \sim 10^{-12}$ and $St_{1000nm} \sim 10^{-8}$ respectively. The Stokes number St≪1 shows that all particles in this study would almost instantaneously align with the flow field and stream in the same direction with the fluid flow. Hence, the particle size does not affect

the particle motion due to fluid flow in our case, and thereby, should not be the cause of the different supraparticle shapes.

S6. Formation of silica shell close to oil-water interface

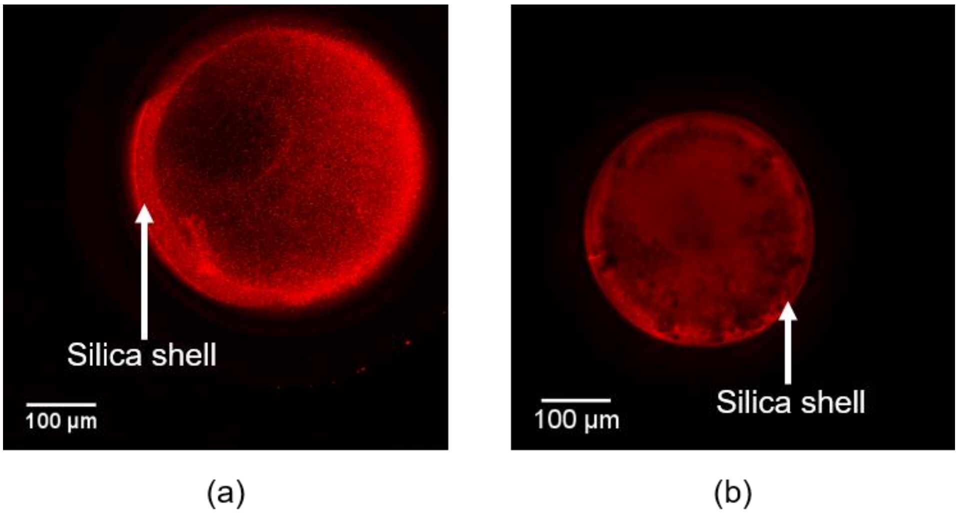

Figure S5. Fluorescence emission signals from the rhodamine labelled silica particles shows the presence of a silica shell close to the oil-water interface for both (a) large (800 nm) and (b) small (30 nm) particles.

S7. Contraction of silica shell

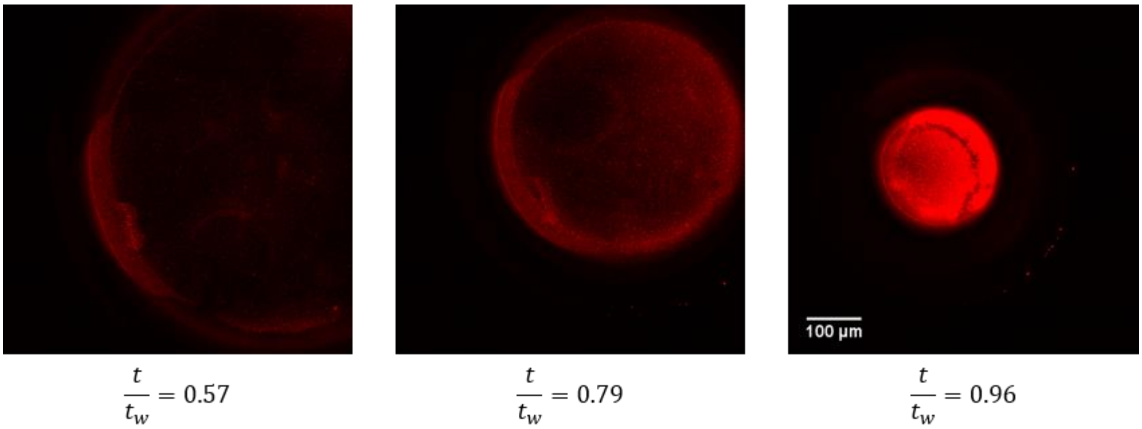

Figure S6. Fluorescence emission signals from the rhodamine labelled silica particles shows that the silica shell keeps on contracting, adjusting its shape to the oil-water interface.

S8. Peclet number calculations

In our case, we can define Peclet number as

$$Pe = \frac{t_{mass\ diffusion}}{t_{interface\ movement}}$$

$$t_{mass\ diffusion} = \frac{l^2}{D}$$

where l is the characteristic length scale and D is the diffusion coefficient given by the Stokes-Einstein relation as

$$D = \frac{kT}{6\pi\mu r_p}$$

For silica shell forming at the oil-water interface, we can take the characteristic length scale to be $l \sim 0.5 * D_{2,@t_w}$

Further,

$$t_{interface\ movement} = \frac{l}{v_{interface,average}}$$

where $v_{interface,average}$ is the average rate at which the oil-water interface is receding, that is calculated using Figure 2e.

Therefore, the Peclet numbers (corresponding to the oil-water interface) for the 1000 nm and 20 nm diameter particles are $Pe_{o/w,1000} \sim 1.6\ x\ 10^3$ and $Pe_{o/w,20} \sim 3.2\ x\ 10^1$ respectively. Similarly, the Peclet number (corresponding to the air-water interface) for 1000 nm and 20 nm diameter particles are $Pe_{a/w,1000} \sim 7.6\ x\ 10^2$ and $Pe_{a/w,20} \sim 1.5\ x\ 10^1$ respectively.

S9. Stability of supraparticles

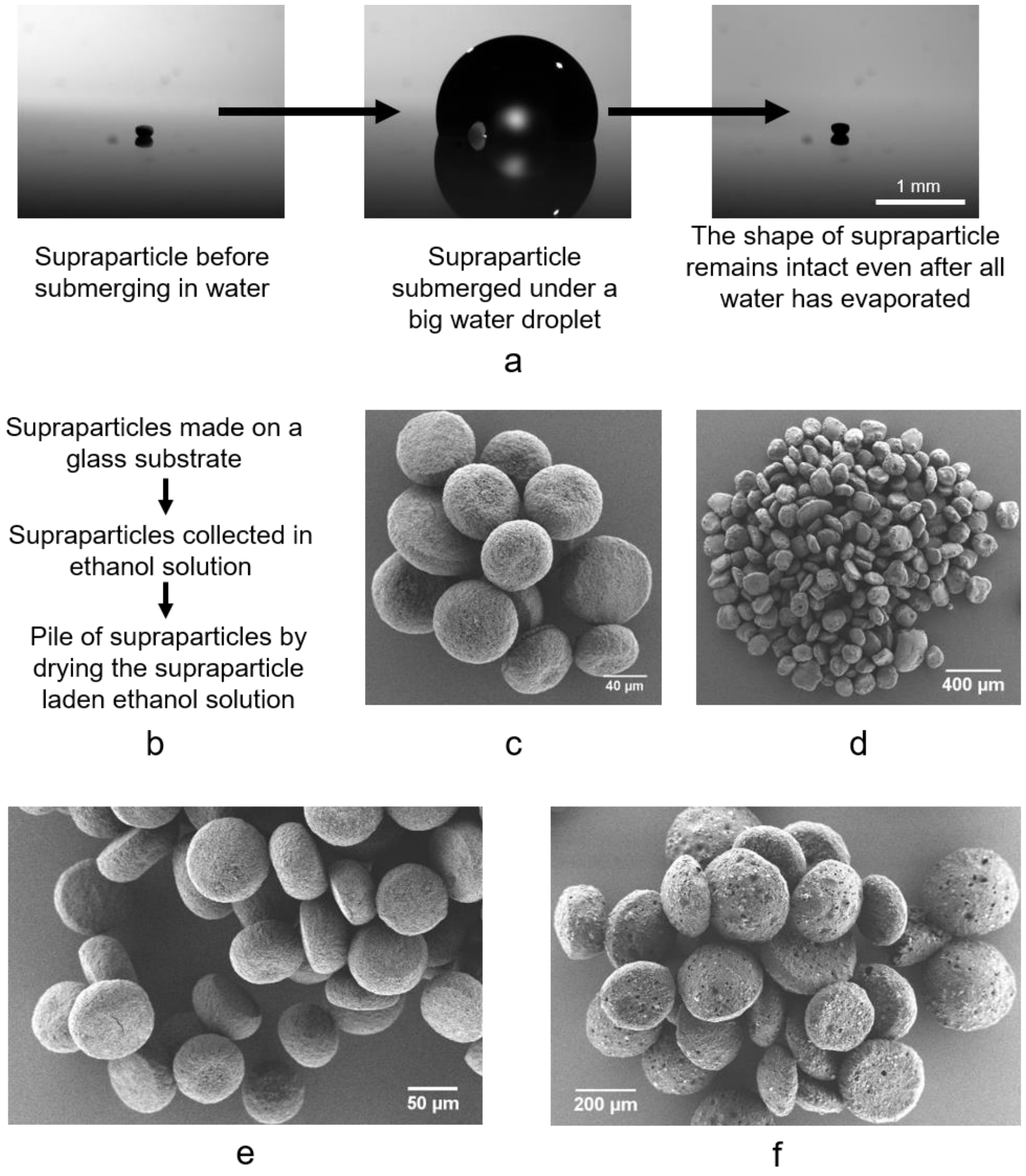

Figure S7. Images showing the stability of supraparticles. (a) Supraparticle made of silica particles of 1000 nm diameter were submerged under a water droplet and recovered intact after all water evaporates. (b) Procedure to collect supraparticles into an ethanol solution. (c)-(f) SEM images of supraparticles made by this method, consisting of (c,e) TiO_2 nanoparticles, (d) tin nanoparticles, (f) mixture of silica and TiO_2 nanoparticles. The nanoparticles used for the experiments in (c) to (f) are commercial nanoparticles, showing the stability of supraparticles even made of commercial nanoparticles.

To show the stability of the supraparticles, a supraparticle, obtained by evaporation of an ouzo droplet containing silica particles of 1000 nm diameter, is submerged under a large water droplet (Figure S7a). The supraparticle remains intact without disintegrating into the constituent nanoparticles. The same is also shown in the Supplementary video (V1). Further, we also show the stability of supraparticles produced by this method, but using commercial nanoparticles, by generating a large amount of supraparticles on a single glass substrate (Figure S7b, Supplementary Video V2). Thereafter, we used ethanol to rinse them off from the surface into a bath. Then we deposited a drop of the supraparticle-suspension on a new surface. The SEM images of these collected pile of supraparticles show that they are stable without disintegrating into the constituent particles (Figure S7c-f).

S10. SEM images of supraparticles

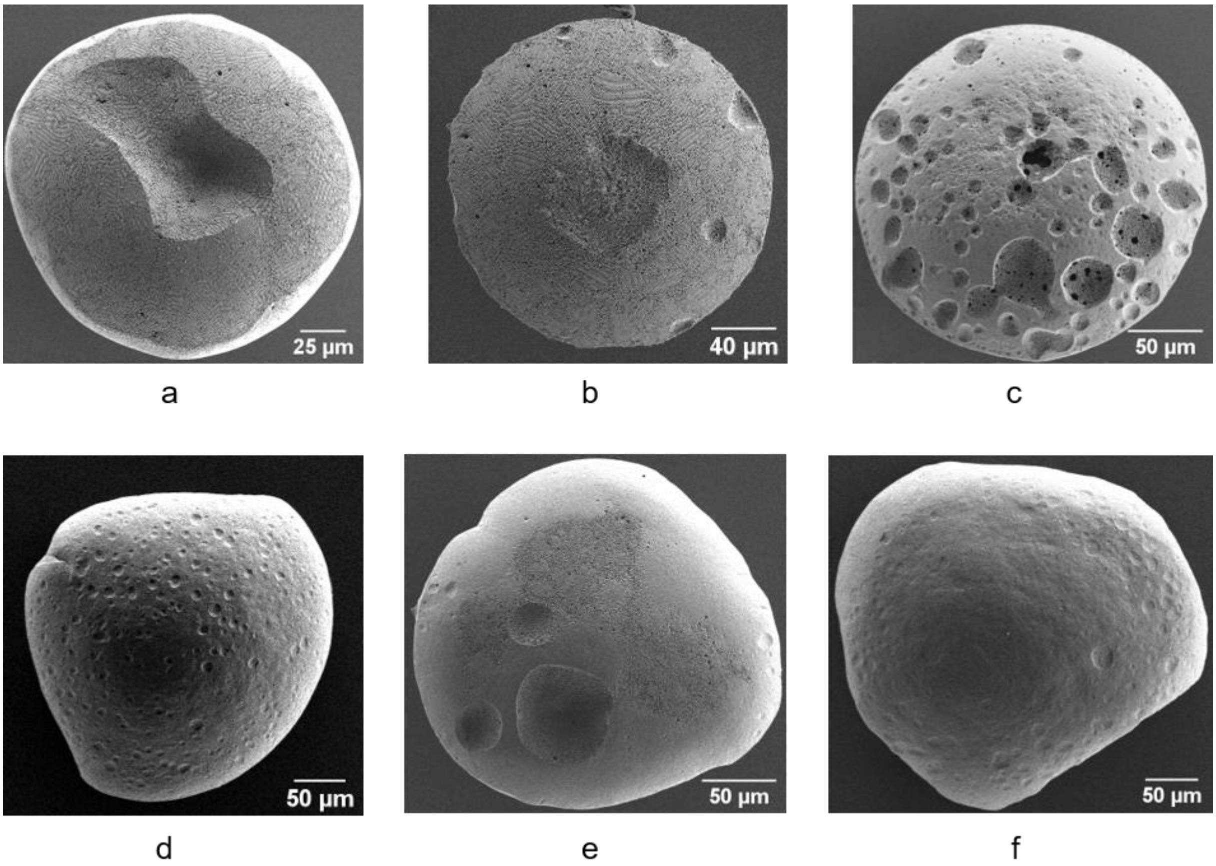

Figure S8. SEM images of supraparticles obtained from silica particles of size (a) 600 nm, (b) 1000 nm, (c) 20 nm (10%)+1000 nm (90%), (d) 20 nm (50%) + 1000 nm (50%), (e) 20 nm (10%) + 600 nm (90%), (f) 20 nm (50%) + 600 nm (50%).

S11. SEM images showing smaller particles on the outer surface of bicolloidal supraparticle

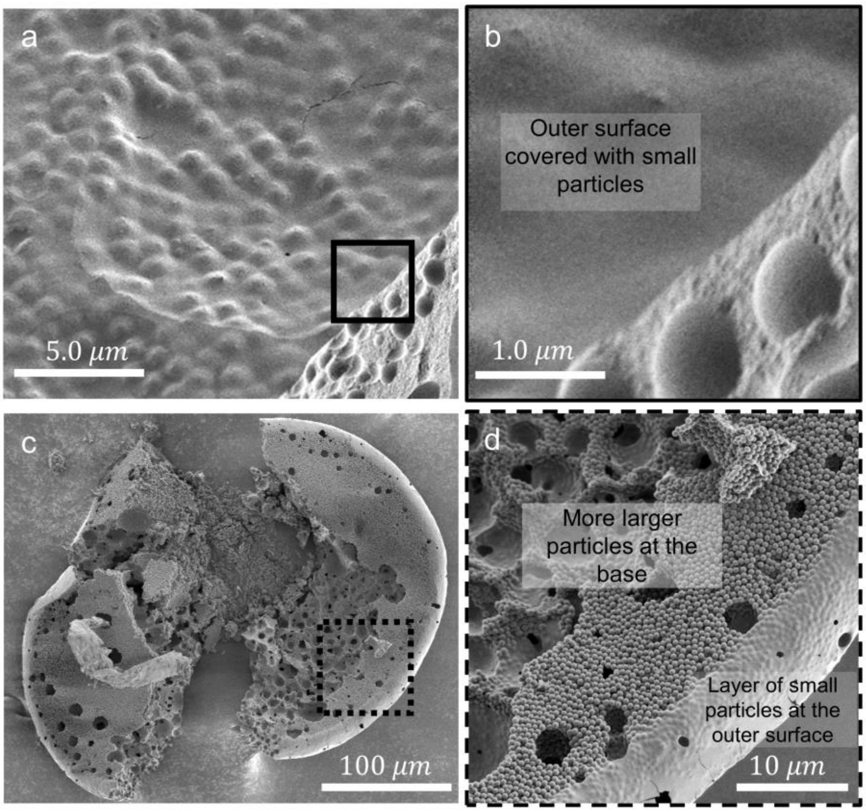

Figure S9. Spatially varying distribution of particles in bicolloidal supraparticles, observed using SEM. These supraparticles are composed of 30 wt% 20 nm diameter silica particles and 70 wt% 1000 nm diameter silica particles. (a) The outer surface of the bicolloidal supraparticle is covered with smaller particles. (b) Magnified image of the rectangular region marked in (a). (c) Bottom side of a bicolloidal supraparticle. (d) Magnified image of the dotted rectangular region in (c), showing a layer of smaller particles at the outer surface, while the central region is rich in larger particles.

S12. Characterization of 1000 nm silica particles by SEM

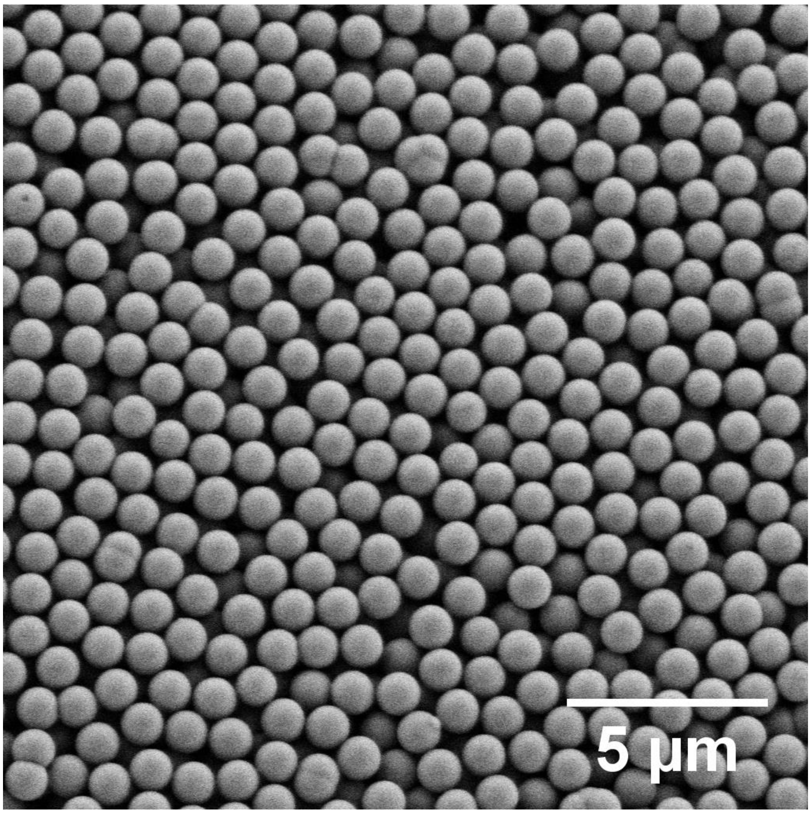

Figure S10. SEM image of silica particles with particle diameter 1000 nm used in this study. The particles have a size of 1056 ± 24 nm. Scale bar: 5 µm.

S13. Plots of droplet evaporation

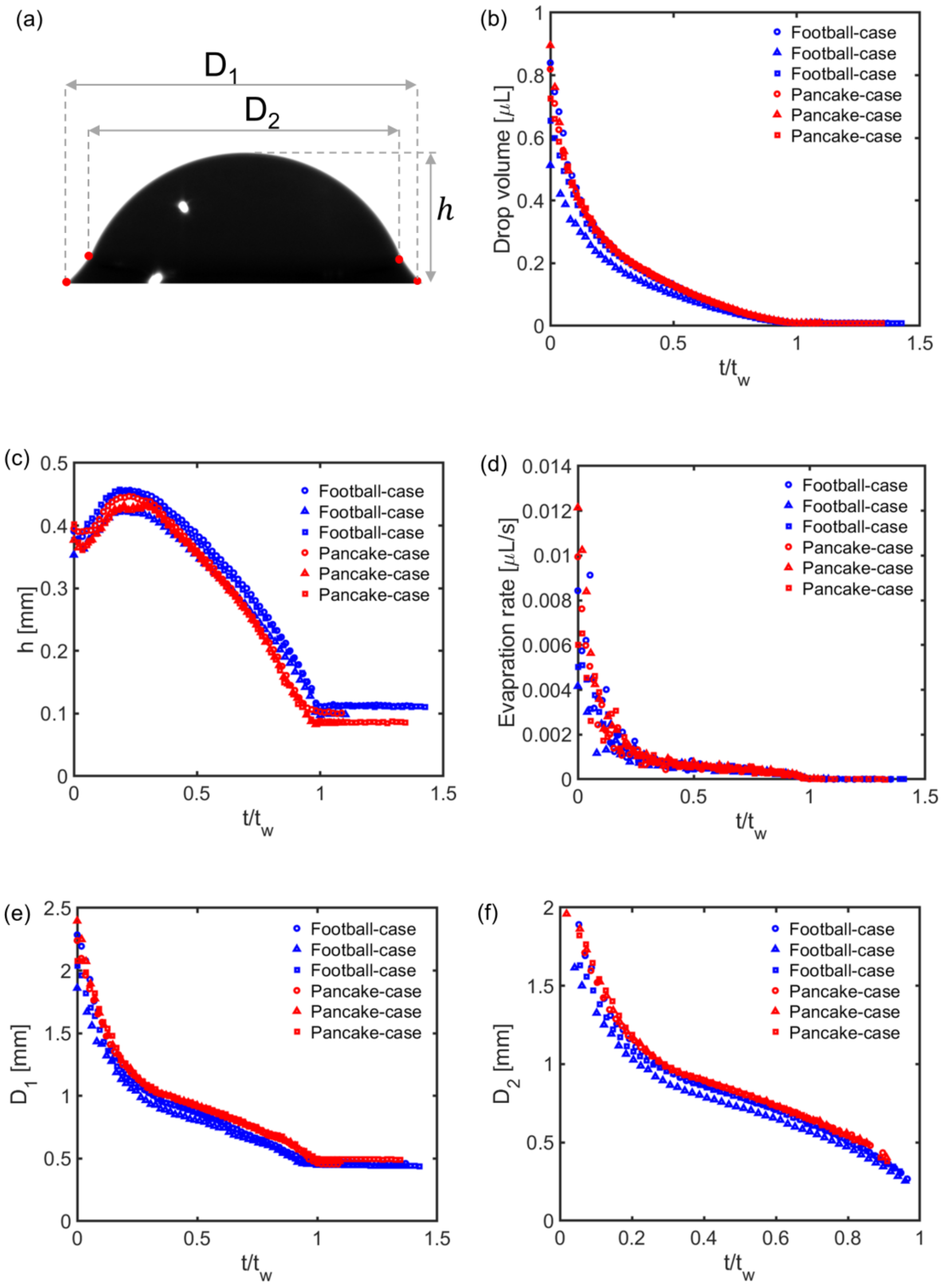

Figure S11. Drop volume, drop height (h), evaporation rate, liquid-solid-air contact-line diameter (D_1), and the estimated oil-water-air contact-line diameter (D_2), plotted *versus* time (normalized to t_w) for "Football case" (1000 nm silica particles, marked in blue) and "Pancake case" (20 nm silica particles, marked in red). t_w = 600±36 s.

S14. Mechanical stability of supraparticles

To test the mechanical stability of the supraparticles, they were compressed using a z-stage that was driven by a stepper motor. The compression tool (CT) is a small glass slide (2x2 mm^2) mounted to a force sensor. The CT is moved towards the supraparticle in steps of 0.5 µm. After each step, the signal from the force sensor is averaged for 0.2 seconds, to obtain the force measured at that position.

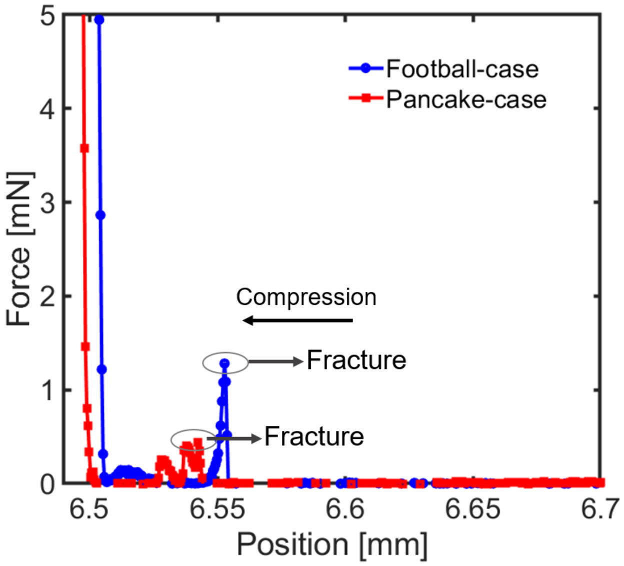

Figure S12 Typical compression-test curves for football-shaped supraparticles (made of 1000 nm silica particles, blue circles) and pancake shaped supraparticles (made of 20 nm silica particles). Position (x-axis) refers to the position of the compression tool, measured relative to an arbitrary reference. As the magnitude of the position decreases, the compression tool comes closer to the supraparticle. The moment when the compression tool (CT) makes contact with the supraparticle, the force starts to increase (close to 6.55 mm for the considered samples). The point where the supraparticles fractures, is marked in the figure, after which the force decreases. The subsequent increase in force close to 6.5 mm is because of the compression of the fractured fragments of the supraparticle.

The football shaped supraparticles (the ones made of 1000 nm silica particles) were observed to fracture at a compressive force of 1.5 ± 0.3 mN, whereas pancake-shaped supraparticles (the ones made of 20 nm silica particles) were observed to fracture at a compressive force of 0.8 ± 0.5 mN.

REFERENCES

1. Diddens, C.; Tan, H.; Lv, P.; Versluis, M.; Kuerten, J. G. M.; Zhang, X.; Lohse, D. Evaporating Pure, Binary and Ternary Droplets: Thermal Effects and Axial Symmetry Breaking. *J. Fluid Mech.* **2017,** *823*, 470-497.
